# Bone metabolism dynamics in the early post-transplant period following kidney and liver transplantation

**DOI:** 10.1371/journal.pone.0191167

**Published:** 2018-01-16

**Authors:** Peter W. Schreiber, Heike A. Bischoff-Ferrari, Katia Boggian, Marco Bonani, Christian van Delden, Natalia Enriquez, Thomas Fehr, Christian Garzoni, Hans H. Hirsch, Cédric Hirzel, Oriol Manuel, Pascal Meylan, Lanja Saleh, Maja Weisser, Nicolas J. Mueller, the Swiss Transplant Cohort Study (STCS)

**Affiliations:** 1 University Hospital Zurich and University Zurich, Division of Infectious Diseases and Hospital Epidemiology, Zurich, Switzerland; 2 University Hospital Zurich and University of Zurich, Department of Geriatrics and Aging Research, Zurich, Switzerland; 3 Cantonal Hospital St. Gallen, Division of Infectious Diseases and Hospital Hygiene, St. Gallen, Switzerland; 4 University Hospital Zurich and University Zurich, Department of Nephrology, Zurich, Switzerland; 5 University Hospitals Geneva and University of Geneva, Department of Surgery, Service of Transplantation, Geneva, Switzerland; 6 Cantonal Hospital Chur, Internal Medicine, Chur, Switzerland; 7 Bern University Hospital (Inselspital), Department of Infectious Diseases, University of Bern, Bern, Switzerland; 8 University Hospital Basel, Division of Infectious Diseases and Hospital Epidemiology, Basel, Switzerland; 9 University Hospital (CHUV) and University of Lausanne, Infectious Diseases Service, Lausanne, Switzerland; 10 University Hospital Zurich, Institute of Clinical Chemistry, Zurich, Switzerland; Medical College of Wisconsin, UNITED STATES

## Abstract

Bone disease contributes to relevant morbidity after solid organ transplantation. Vitamin D has a crucial role for bone metabolism. Activation of vitamin D depends on the endocrine function of both, liver and kidney. Our study assessed key markers of bone metabolism at time of transplantation and 6 months after transplantation among 70 kidney and 70 liver recipients. In 70 kidney recipients 25-OH vitamin D levels did not differ significantly between peri-transplant (median 32.5nmol/l) and 6 months post-transplant (median 41.9nmol/l; *P* = 0.272). Six months post-transplant median 1, 25-(OH)_2_ vitamin D levels increased by >300% (from 9.1 to 36.5ng/l; *P*<0.001) and median intact parathyroid hormone levels decreased by 68.4% (from 208.7 to 66.0 ng/l; *P*<0.001). Median β-Crosslaps (CTx) and total procollagen type 1 amino-terminal propeptide (P1NP) decreased by 65.1% (from 1.32 to 0.46ng/ml; *P*<0.001) and 60.6% (from 158.2 to 62.3ng/ml; *P*<0.001), respectively. Kidney recipients with incident fractures had significantly lower levels of 1, 25-(OH)_2_ vitamin D at time of transplantation and of intact parathyroid hormone 6 months post-transplant. Among 70 liver recipients, 25-OH vitamin D, 1, 25-(OH)_2_ vitamin D and intact parathyroid hormone levels were not significantly altered between peri-transplant and 6 months post-transplant. Contrary to kidney recipients, median CTx increased by 60.0% (from 0.45 to 0.72 ng/ml; *P* = 0.002) and P1NP by 49.3% (from 84.0 to 125.4ng/ml; *P* = 0.001) in the longitudinal course. Assessed biomarkers didn’t differ between liver recipients with and without fractures. To conclude, the assessed panel of biomarkers proved highly dynamic after liver as well as kidney transplantation in the early post-transplant period. After kidney transplantation a significant gain in 1, 25-(OH)_2_ vitamin D combined with a decline in iPTH, CTx and P1NP, whereas after liver transplantation an increase in CTx and P1NP were characteristic.

## Introduction

Solid organ transplantation is an established treatment for patients with end-stage renal failure or liver insufficiency. In the US more than 19’000 kidney and 7’000 liver transplantations were performed in 2016 [1]. Bone disease and resulting fractures are an important co-morbidity in patients with end-stage organ disease [2, 3].

In fact, it has been shown that the majority of liver recipients has abnormal bone mineral density (BMD) already at time of transplantation or has suffered from fractures pre-transplant [2, 4]. This may be explained by excessive alcohol consumption in some of these patients [5], but hyperbilirubinemia [6], hypogonadism [7] and reduced insulin-like growth factor-1 levels [8] may also contribute to abnormal bone metabolism. Among patients with end-stage renal failure, bone health is impaired with renal osteodystrophy presenting either as osteitis fibrosa, osteomalacia, adynamic bone disease or a mixed type [9, 10]. Consequently, both kidney and liver transplant patients have been found to have a high risk of bone loss and fractures [11, 12].

Bone mineralization depends on adequate calcium and phosphate levels [13, 14]. Two pivotal hormones regulating these minerals are 1, 25-(OH)_2_ vitamin D (1, 25-(OH)_2_D) and parathyroid hormone (PTH). The most important source of vitamin D is synthesis in the epidermis via ultraviolet B exposure. Vitamin D undergoes a first hydroxylation at the 25-position in the liver resulting in 25-OH vitamin D (25-OHD). 25-OHD is still a precursor of the active hormone, but due to its long half-life of 2 to 3 weeks it best reflects vitamin D status [15]. The circulating active hormone, 1, 25-(OH)_2_D, emerges from 25-OHD via an additional hydroxylation at the 1-position in the kidney. 1, 25-(OH)_2_D increases calcium levels not only by stimulation of intestinal calcium resorption but also in corroboration with PTH by increased renal resorption and mobilization out of bone tissue. Furthermore, 1, 25-(OH)_2_D increases intestinal phosphate absorption and decreases phosphate excretion via the kidneys. Main drivers for production of 1, 25-(OH)_2_D are PTH or hypophosphatemia, whereas calcium, fibroblast growth factor 23 and the active hormone 1, 25-(OH)_2_D itself are inhibitory [13, 16]. PTH excretion is suppressed by 1, 25-(OH)_2_D and a greater calcium intake [17].

Despite the substantial morbidity caused by impaired bone health among transplant patients, detailed data on bone metabolism changes after kidney or liver transplantation—both key organs of vitamin D hydroxylation—is limited. In the current study we used prospectively collected samples for measurements of vitamin D metabolites (25-OHD, 1, 25-(OH)_2_D), intact PTH (iPTH), creatinine and two bone turnover markers, β-Crosslaps (CTx) and total procollagen type 1 amino-terminal propeptide (P1NP), in the same patients at time of transplantation and 6 months after transplantation. In line with recent recommendations CTx was used as parameter for bone resorption and P1NP for bone formation [18, 19]. We additionally reviewed all medical records for dual energy x-ray absorptiometry (DXA) scans performed, incident fractures recorded during post-transplant routine care, and supplementation of vitamin D and calcium.

## Materials and methods

### Study design, population and patient-related data

This study was a nested project within the Swiss Transplant Cohort Study (STCS, www.stcs.ch). Since May 2008 data on all solid organ transplants carried out in Switzerland have been prospectively collected in the STCS database [20]. All Swiss transplant centers, i.e. Basel, Bern, Geneva, St. Gallen, Lausanne and Zurich, contribute to data acquisition. The STCS was approved by the Ethic Committees of all participating institutions, i.e. Ethikkommission Nordwest- und Zentralschweiz EKNZ, Ethikkommission Bern, Ethikkommission Genf, Ethikkommission Ostschweiz EKOS, Ethikkommission Zürich. None of the transplant donors were from a vulnerable population and all donors or next of kin provided written informed consent that was freely given". Information about vitamin D supplementation, calcium supplementation, BMD measurements and incidence of fractures were retrospectively collected by patient chart review, whereas all other data derived from prospective records. DXA scans to determine BMD were performed at the discretion of the treating physician. All fractures were radiographically confirmed. 70 kidney and 70 liver recipients were analyzed. Kidney transplantations and liver transplantations were performed between 05.05.2008 to 28.09.2009 and between 16.05.2008 to 20.12.2009, respectively. Median follow up was 5.6 years (IQR 5.5–5.8 years) in kidney recipients and 4.9 years (IQR 4.6–5.4 years) in liver graft recipients. For the subgroup analysis of female transplant recipients a simplified age-based approach was chosen to define menopausal status [21].

### Laboratory analysis

For laboratory analyses prospectively collected citrate plasma samples, drawn at the time of transplant and 6 months post-transplant, which were stored at -80°C in the STCS biobank. These samples were retrieved from the STCS biobank for centralized, uniform measurement at the Institute of Clinical Chemistry of the University Hospital Zurich. 25-OHD measurement was performed with Roche Diagnostics Vitamin D total assay on Cobas 8000 (Roche Diagnostics, Mannheim, Germany). Vitamin D status was categorized as follows: 25-OH vitamin D < 25nmol/l severe deficiency, ≥ 25 and < 50nmol/l deficiency and ≥ 50nmol/l no deficiency.

1, 25-(OH)_2_D was determined with IDS-iSYS 1, 25-Di(OH)D on IDS-iSYS Multi-Discipline Automated System (Immunodiagnostic Systems Holdings PLC, Tyne and Wear, United Kingdom).

Intact PTH, CTx and total P1NP were measured using Roche Diagnostics Elecsys PTH (1–84) test, β–Crosslaps/serum and total P1NP on Cobas 8000 (Roche Diagnostics, Mannheim, Germany), respectively.

Creatinine was determined with a kinetic color test based on Jaffe Method from Roche Diagnostics (Mannheim, Germany) running on Cobas c701 system. Estimated glomerular filtration rate (eGFR) was calculated according to the CKD-EPI method [22], as this method was shown to provide more reliable results after liver transplantation [23].

Phosphate concentrations in plasma samples was measured using Roche Phosphat Molybdate assay (PHOS2) running on Cobas 8000 System (Roche Diagnostics, Mannheim, Germany).

### Statistical analysis

All statistical analyses were performed with R (version 3.2.3). Continuous variables were reported as median and interquartile range (IQR), categorical variables as absolute numbers and frequencies (%). Statistical testing was performed with two-sided tests, p-values < 0.05 were considered significant. Wilcoxon rank-sum test was used for comparison of continuous variables between two groups, whereas Wilcoxon matched-pairs signed-rank test was applied for pairwise comparisons. Categorical variables were compared with Fisher’s exact test. For investigation of linear relationships between two variables linear regression was used.

## Results

### Patients’ characteristics

A total of 140 consecutive patients, 70 kidney and 70 liver transplant recipients, were included in this study. Baseline characteristics are shown in Table 1.

**Table 1:** Baseline characteristics.

	kidney (n = 70)	liver (n = 70)
**Age median (IQR)**	52y (39, 62)	55y (43, 62)
**Sex**	Male 42 (60%)	Male 47 (67%)
	Female 28 (40%)^¶^	Female 23 (33%)^¶^
	Premenopausal 20	Premenopausal 15
	Postmenopausal 8	Postmenopausal 8
**Ethnicity**	Caucasian 62 (88.6%)	Caucasian 69 (98.6%)
	African 4 (5.7%)	African 1 (1.4%)
	Asian 3 (4.3%)	
	American Indian 1 (1.4%)	
**Underlying disease**	Glomerulonephritis 16 (22.9%)	Chemical cirrhosis 18 (25.7%)
	Polycystic kidney disease 12 (17.1%)	Hepatocellular carcinoma 12 (17.1%)
	Nephrosclerosis 11 (15.7%)	Hepatitis C 10 (14.3%)
	Diabetic nephropathy 7 (10%)	Hepatitis B 7 (10%)
	Reflux nephropathy 4 (5.7%)	Cholangicarcinoma 4 (5.7%)
	Other 20 (28.6%)	Other 19 (27.1%)
**Diabetes mellitus**^¶¶^	24 (34.3%)	29 (41.4%)
**Renal replacement therapy**	HD: 43 (61.4%)	
	PD: 16 (22.9%)	
	None: 11 (15.7%)	
**Hepatorenal syndrome**		present, no RRT 9 (12.9%)
		present, RRT 6 (8.6%)
		absent, no RRT 51 (72.9%)
		unknown 4 (5.7%)
**Type of donation**	DBD 32 (45.7%)	DBD 64 (91.4%)
	living related 19 (27.2%)	living related 5 (7.1%)
	living unrelated 19 (27.2%)	living unrelated 1 (1.4%)
**Type of transplant**		Whole liver 65 (92.9%)
		Split right 5 (7.1%)
**Corticosteroid-containing**	Yes 62 (88.6%)	Yes 37(52.9%)
immunosuppression*	No 8 (11.4%)	No 33 (47.1%)
**Vitamin D supplementation**^#^	peri-transplant	peri-transplant
	cholecalciferol 26 (37.1%)	cholecalciferol 8 (11.4%)
	1, 25-dihydroxycholecalciferol 25 (35.7%)**	1, 25-dihydroxycholecalciferol 2 (2.9%)
	paricalcitol 1 (1.4%)	
	6 months post-transplant	6 months post-transplant
	cholecalciferol 49/70 (70.0%)	cholecalciferol 29 (41.4%)
	1, 25-dihydroxycholecalciferol 4 (5.7%)***	1, 25-dihydroxycholecalciferol 1 (1.4%)
**Calcium supplementation**^##^	peri-transplant	peri-transplant
	Yes 53 (75.7%)	Yes 8 (11.4%)
	6 months post-transplant	6 months post-transplant
	Yes 42 (60.0%)	Yes 32 (45.7%)

^¶^ Age-based assignment: <55y premenopausal, ≥55y postmenopausal

^¶¶^ Diagnosis of Diabetes mellitus either already established at time of transplantation or within the first 6 months after transplantation

*at 6 months post-transplant

**1 individual receiving supplementation with cholecalciferol and 1, 25-dihydroxycholecalciferol

***2 individuals receiving supplementation with cholecalciferol and 1, 25-dihydroxycholecalciferol

^#^Median dose of cholecalciferol 800IU (IQR 600–800), median dose of 1, 25-dihydroxycholecalciferol 0.25μg (IQR 0.25–0.25 μg)

^##^Median dose of calcium 1000mg (IQR 806–1200)

Abbreviations: DBD: donation after brain death, HD: hemodialysis, IQR: interquartile range, PD: peritoneal dialysis, RRT: renal replacement therapy

#### Kidney recipients

70 kidney transplant recipients, 60% (n = 42) males, with a median age of 51 years participated in the study (Table 1). Most common causes of chronic renal failure were glomerulonephritis (n = 16, 22.9%), polycystic kidney disease (n = 12, 17.1%), nephrosclerosis (n = 11, 15.7%) and diabetic nephropathy (n = 7, 10%). Cadaveric renal grafts were used in 32 (45.7%) recipients, whereas 38 (54.3%) participants received grafts derived from living donation.

#### Liver recipients

Median age of the enrolled 70 liver recipients was 55 years, 67% (n = 47) were male (Table 1). The majority of liver transplantations was due to chemical cirrhosis (n = 18, 25.7%), hepatocellular carcinoma (n = 12, 17.1%), hepatitis C (n = 10, 14.3%) and hepatitis B (n = 7, 10%). Most participants were transplanted with cadaveric grafts (n = 64, 91.4%).

### 25-OH vitamin D and 1, 25-(OH)_2_ vitamin D

#### Kidney recipients

**Fig 1:**
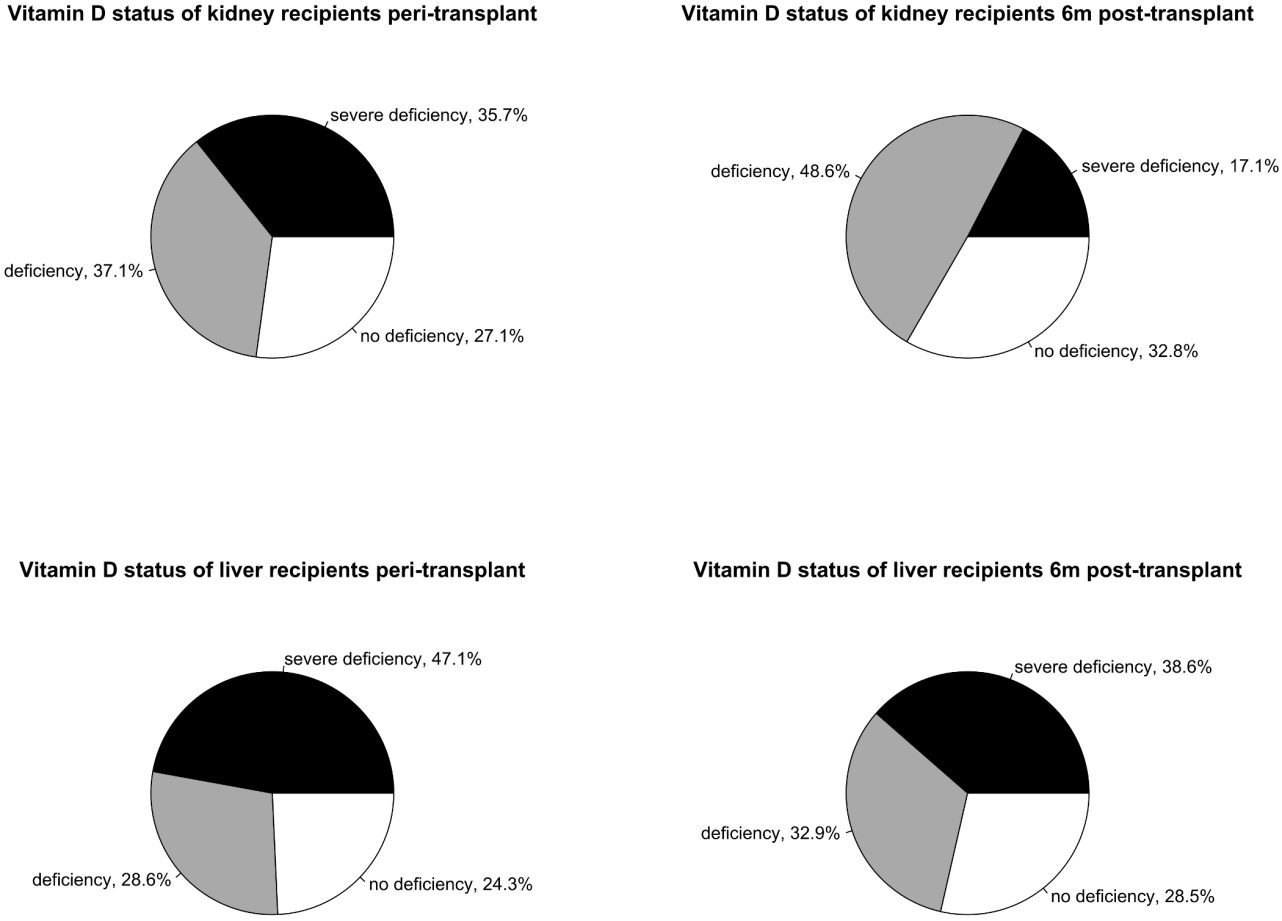
Vitamin D status of kidney recipients (top) and liver recipients (bottom) peri-transplant and 6 months post-transplant.

At time of transplantation the majority of kidney recipients was severely vitamin D deficient (25-OHD < 25 nmol/l; n = 25, 35.7%) or vitamin D deficient (25-OHD ≥ 25 and < 50 nmol/l; n = 26, 37.1%). Vitamin D levels of at least 50 nmol/l were less frequent (n = 19, 27.1%) (Fig 1). Peri-transplant median 1, 25-(OH)_2_D was 9.1 ng/l (IQR 7.5–13.8) (Table 2).

**Table 2:** Measurement of 25-OHD, 1, 25-(OH)_2_D, 1, 25-(OH)_2_D/25OHD ratio, iPTH, CTx, P1NP, creatinine and phosphate peri-transplant and 6 months post-transplant.

	peri-transplant	6 months post-TPL	P*
**kidney**	**n = 70**	**n = 70**	
**25-OHD**	32.5 (18.0–52.0)	41.9 (27.2–53.7)	0.272
**1, 25-(OH)_2_D**	9.1 (7.5–13.8)	36.5 (24.9–48.1)	<0.001
**1, 25-(OH)_2_D/25-OHD**	0.35 (0.19–0.69)	0.87 (0.67–1.36)	<0.001
**iPTH**	208.7 (109.7–338.8)	66.0 (49.2–102.7)	<0.001
**CTx**	1.32 (0.60–2.01)	0.46 (0.20–0.82)	<0.001
**P1NP**	158.2 (93.9–310.8)	62.3 (32.9–105.5)	<0.001
**creatinine**	646.0 (491.2–782.8)	116.5 (102.0–157.5)	<0.001
**eGFR**	7.6 (5.7–10.6)	54.1 (39.3–69.6)	<0.001
**phosphate**	1.48 (1.19–1.87)	0.86 (0.68–1.09)	<0.001
**liver**	**n = 70**	**n = 70**	
**25-OHD**	28.0 (13.1–48.8)	32.3 (17.7–54.1)	0.414
**1, 25-(OH)_2_D**	25.9 (14.8–34.7)	29.7 (18.7–40.7)	0.179
**1, 25-(OH)_2_D/25-OHD**	0.88 (0.58–1.53)	0.85 (0.60–1.41)	0.603
**iPTH**	34.7 (22.6–61.7)	44.9 (35.5–60.6)	0.107
**CTx**	0.45 (0.25–0.81)	0.72 (0.47–1.03)	0.002
**P1NP**	84.0 (53.9–146.4)	125.4 (67.0–200.5)	0.001
**creatinine**	77.0 (62.5–116.5)	99.5 (80.5–130.0)	0.005
**eGFR**	89.1 (55.3–108.9)	65.5 (53.0–84.3)	0.011
**phosphate**	1.06 (0.86–1.34)	1.21 (1.09–1.39)	0.006

Numeric variables expressed as median (IQR).

25-OHD (25-OH vitamin D) reported in nmol/l

1, 25-(OH)_2_D (1, 25-(OH)_2_ vitamin D) reported in ng/l

1, 25-(OH)_2_D/25OHD ratio in ng/nmol

iPTH (intact parathyroid hormone) reported in ng/l

CTx (β-Crosslaps) reported in ng/ml

P1NP (total procollagen type 1 amino-terminal propeptide) reported in ng/ml

Creatinine reported in μmol/l

eGFR (estimated glomerular filtration rate) reported in ml/min/1.73m^2^ (calculated according to CKD-EPI)

Phosphate reported in mmol/l

* Wilcoxon matched-pairs signed-rank test was used for comparison.

**Fig 2:**
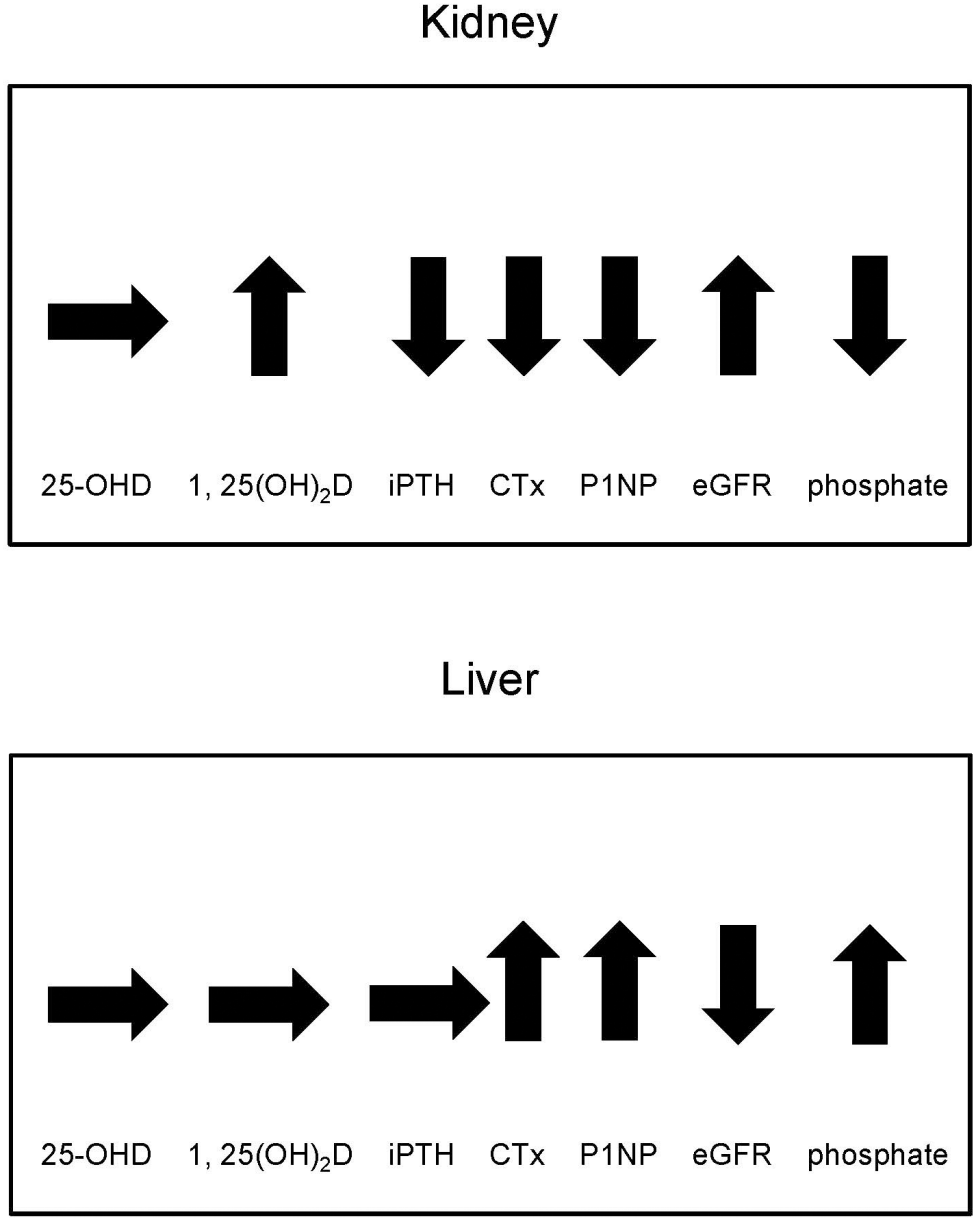
Longitudinal changes in 25-OH vitamin D, 1, 25-(OH)_2_ vitamin D, intact parathyroid hormone, β-Crosslaps, total procollagen type 1 amino-terminal propeptide, estimated glomerular filtration rate, phosphate in the first 6 months after kidney and liver transplantation. Arrows pointing upwards indicate significant increase, arrows pointing downwards indicate significant decrease, horizontal arrows respond to no significant changes. 25-OHD: 25-OH vitamin D, 1, 25-(OH)_2_D: 1, 25-(OH)_2_ vitamin D, iPTH: intact parathyroid hormone, CTx: β-Crosslaps, P1NP: total procollagen type 1 amino-terminal propeptide.

At 6 months post-transplant the number of severely vitamin D deficient patients dropped (n = 12, 17.1%), but vitamin D deficiency remained common (n = 34, 48.6%) (Fig 1). No vitamin D deficiency was measured in 23 (32.8%) participants (1 measurement failed). Six months after transplantation 25-OHD levels were ≥ 50 nmol/l in 37.3% of patients receiving supplementation therapy with a median dose of 800IU/d (vs. 0% in kidney recipients without supplementation therapy). No significant difference in 25-OHD levels between time of transplantation (median 32.5 nmol/l, IQR 18.0–52.0) and 6 months post-transplant (median 41.9 nmol/l, IQR 27.2–53.7) was detected (*P* = 0.272) (Fig 2). 6 months post-transplant 1, 25-(OH)_2_D (median 36.5 ng/l, IQR 24.9–48.1) was significantly higher than peri-transplant (*P*<0.001) (Table 2).

The ratio of the active hormone 1, 25-(OH)_2_D to its inactive precursor 25-OHD increased from peri-transplant (median 0.35 ng/nmol, IQR 0.19–0.69) to 6 months post-transplant (median 0.87 ng/nmol, IQR 0.67–1.36; *P*<0.001).

#### Liver recipients

In most liver transplant recipients severely deficient (n = 33, 47.1%) or deficient (n = 20, 28.6%) 25-OHD levels were detected at time of transplantation (Fig 1). A minor proportion of liver recipients showed no vitamin D deficiency (n = 17, 24.3%). Median 1, 25-(OH)_2_D was 25.9 nmol/l (IQR 14.8–34.7) peri-transplant (Table 2).

Six months after transplantation the majority of liver recipients had severe 25-OHD deficiency (n = 27, 38.6%) or deficiency (n = 23, 32.9%). 25-OHD levels of at least 50nmol/l were detected in 20 patients (28.5%) (Fig 1). No vitamin D deficiency was detected in 36.7% of liver recipients with supplementation therapy (median dose 800IU/d) and 23.1% without supplementation therapy. 25-OHD levels remained stable between measurement peri- and 6 months post-transplant (*P* = 0.414) (Fig 2). Six months post-transplant 1, 25-(OH)_2_D levels did not differ significantly from peri-transplant levels (median 29.7 nmol/l, IQR 18.7–40.7; *P* = 0.179) (Table 2). Similarly, no significant difference in the ratio of 1, 25-(OH)_2_D to 25-OHD was detectable between measurement peri-transplant (median 0.88 ng/nmol, IQR 0.58–1.53) and 6 months post-transplant (median 0.85 ng/nmol, IQR 0.60–1.41; *P* = 0.603).

### Estimated glomerular filtration rate, phosphate, intact parathyroid hormone, β-Crosslaps and total procollagen type 1 amino-terminal propeptide

#### Kidney recipients

At time of transplantation median eGFR was 7.6 ml/min/1.73m^2^ (IQR 5.7–10.6); 6 months post-transplant eGFR was remarkably improved (median 54.1 ml/min/1.73m^2^, IQR 39.3–69.6) indicating excretory function of the kidney graft (*P*<0.001) (Table 2). Phosphate levels decreased from 1.48 (IQR 1.19–1.87) to 0.6 mmol/l (IQR 0.68–1.09) in the longitudinal course (*P*<0.001). Median iPTH was 208.7 ng/l (IQR 109.7–338.8) peri-transplant and dropped significantly to a median of 66.0 ng/l (IQR 49.2–102.7) 6 months post-transplant (*P*<0.001). CTx levels, reflecting bone resorption, measured peri-transplant (median 1.32 ng/ml, IQR 0.60–2.01) were significantly higher than 6 months post-transplant (median 0.46 ng/ml, IQR 0.20–0.82; *P*<0.001). Similarly, P1NP values decreased from peri-transplant (median 158.2 ng/ml, IQR 93.9–310.8) to 6 months post-transplant (median 62.3 ng/ml, IQR 32.9–105.5, *P*<0.001). CTx and P1NP were positively correlated at time of transplantation (R^2^ = 0.46, *P*<0.001) as well as 6 months post-transplant (R^2^ = 0.38, *P*<0.001) (S1 Fig). Similarly, CTx and iPTH showed a linear relationship peri-transplant (R^2^ = 0.13, *P* = 0.002) and 6 months post-transplant (R^2^ = 0.30, P<0.001) (S2 Fig).

#### Liver recipients

Estimated glomerular filtration rate showed a significant decrease from peri-transplant (median 89.1 ml/min/1.73m^2^, IQR 55.3–108.9) to 6 months post-transplant (median 65.5 ml/min/1.73m^2^, IQR 53.0–84.3; *P* = 0.011) (Table 2). Median phosphate was 1.06 mmol/l (IQR 0.86–1.34) at time of transplantation and increased to 1.21 mmol/l (1.09–1.39) at 6 months post-transplant (*P* = 0.006). No remarkable difference was observed between peri-transplant and 6 months post-transplant iPTH levels. Peri-transplant CTx levels were significantly lower (median 0.45 ng/ml, IQR 0.25–0.81) than 6 months post-transplant (median 0.72 ng/ml, IQR 0.47–1.03; *P* = 0.002). P1NP levels also increased between measurement peri-transplant (median 84.0 ng/ml, IQR 53.9–146.4) and 6 months post-transplant (median 125.4 ng/ml, IQR 67.0–200.5; *P* = 0.001). Like in kidney transplantation, a positive correlation between CTx and P1NP (peri-transplant R^2^ = 0.25, *P*<0.001; 6 months post-transplant R^2^ = 0.46, *P*<0.001) (S1 Fig) as well as between CTx and iPTH (peri-transplant R^2^ = 0.21, *P*<0.001; 6 months post-transplant R^2^ = 0.24, *P*<0.001) (S2 Fig) was detectable.

### Incident fractures and bone densitometry

#### Kidney recipients

**Fig 3:**
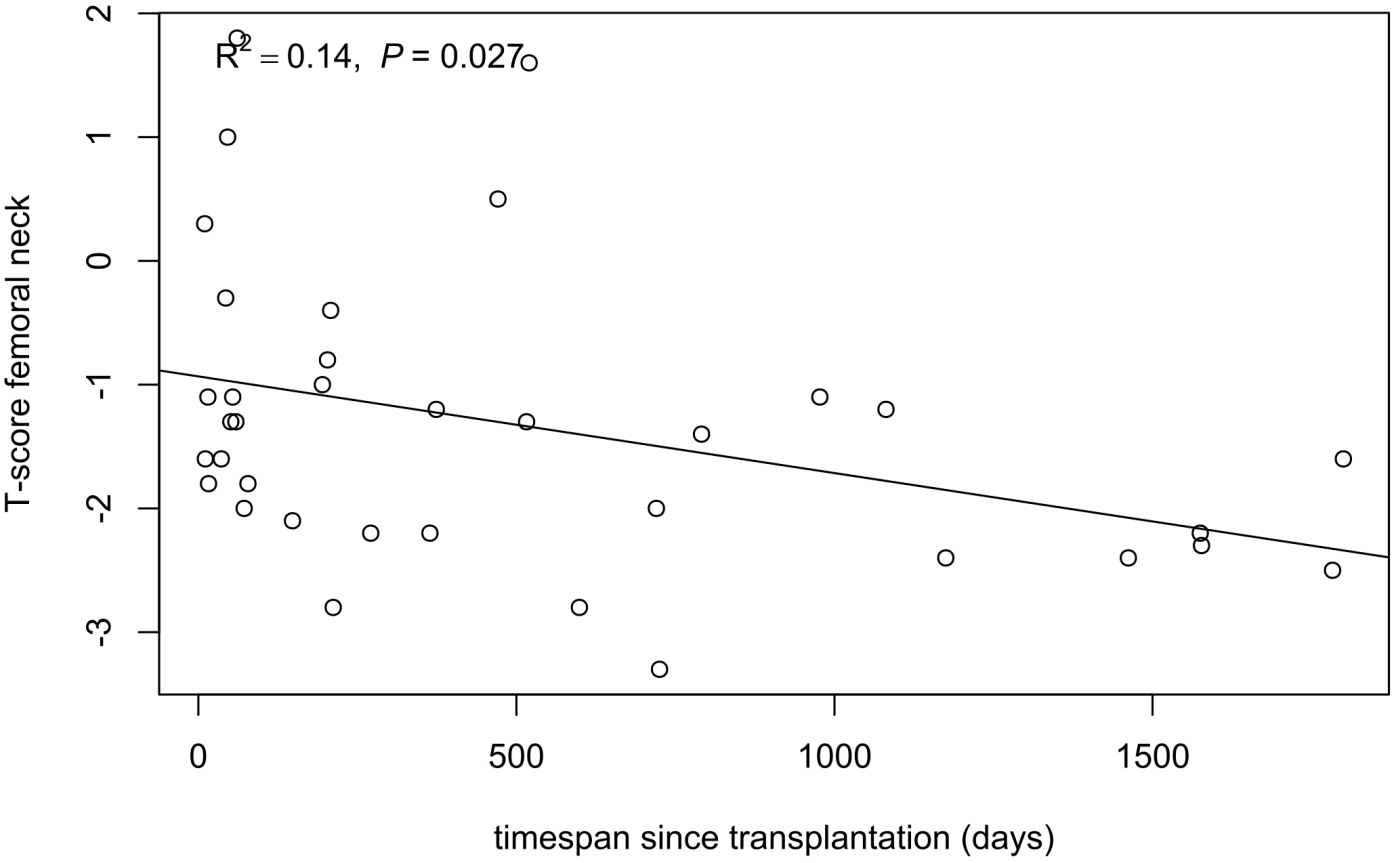
Linear relationship between T-score of the femoral neck and post-transplant timespan in kidney recipients. Line was generated corresponding to univariable linear regression.

Overall 7 fractures occurred in a total of 7 kidney recipients (corresponding to an incidence of 16 fractures per 1000 person-years); 4 (57.1%) fractures affected the lower extremities, 2 (28.6%) the upper extremities and 1 (14.3%) the spine. The median time interval from transplantation to fracture was 866 days (IQR 352–1368). Kidney recipients with incident fractures had lower 1, 25-(OH)_2_D levels peri-transplant (*P* = 0.008) (Table 3). Likely, 6 months post-transplant 1, 25-(OH)_2_D tended to be higher in kidney recipients without incident fractures (*P* = 0.089). Patients suffering from incident fractures showed a marginally significant better eGFR (*P* = 0.049) at time of transplantation, lower iPTH levels (*P* = 0.008) and a trend of lower CTx levels (*P* = 0.064) 6 months after transplantation. BMI tended to be higher in patients without incident fractures (*P* = 0.054). In 36 of 70 kidney recipients DXA scans were available. As bone densitometry was performed by the treating physician’s indication, a large variety in timespan from transplant to bone densitometry was observed (median 242 days, IQR 57–742). Osteoporosis, defined by a T-score of less than -2.5 at any location, was present in 9 (25%) and osteopenia (T-score ≥ − 2.5 and < − 1) in 20 (55.6%) individuals, whereas only 7 (19.4%) kidney transplant recipients had normal BMD. Notably, an increasing timespan until DXA scan was associated with a linear decrease in the T-score of the femoral neck (R^2^ = 0.14, *P* = 0.027) (Fig 3). In 5 individuals with incident fractures bone densitometry was available. Fractures were recorded in 1 patient with osteoporosis, 3 patients with osteopenia and 1 patient with normal BMD.

**Table 3:** Comparison between kidney recipients and liver recipients with incident fractures and without fractures, respectively.

	Fracture	Non-fracture	P*
**kidney**	**n = 7**	**n = 63**	
**Sex (male)**	3 (42.9%)	39 (61.9%)	0.426
**Age**	48.4 (43.6–54.7)	52.7 (38.4–62.2)	0.984
**BMI**	19.7 (18.5–25.1)	24.7 (22.8–27.6)	0.054
**Steroid containing immunosuppressive regimen**^¶^	7 (100%)	55 (87.3%)	1
**Diabetes mellitus**	2 (28.6%)	22 (34.9%)	1
**25-OHD peri-transplant**	30.0 (13.7–37.2)	34.4 (18.7–52.9)	0.318
**25-OHD 6 months post-transplant**	56.7 (44.3–61.7)	41.2 (27.2–52.7)	0.176
**1, 25-(OH)**_**2**_**D peri-transplant**	< 7.5^#^	9.3 (7.5–14.6)	0.008
**1, 25-(OH)**_**2**_**D 6 months post-transplant**	26.0 (17.2–30.6)	38.1 (25.7–48.3)	0.089
**iPTH peri-transplant**	115.8 (111.3–170.0)	216.2 (108.8–351.8)	0.248
**iPTH 6 months post-transplant**	42.6 (38.1–46.5)	69.7 (54.6–106.2)	0.008
**CTx peri-transplant**	0.58 (0.52–1.64)	1.44 (0.68–2.01)	0.225
**CTx 6 months post-transplant**	0.30 (0.13–0.40)	0.52 (0.23–0.85)	0.064
**P1NP peri-transplant**	113.0 (48.1–263.0)	165.3 (94.4–317.9)	0.253
**P1NP 6 months post-transplant**	56.3 (45.7–86.6)	63.1 (32.4–108.7)	0.741
**eGFR peri-transplant**	10.7 (8.6–20.2)	7.1 (5.7–10.1)	0.049
**eGFR 6 months post-transplant**	52.1 (48.9–63.8)	54.3 (37.9–69.5)	0.626
**Phosphate peri-transplant**	1.57 (1.19–2.62)	1.48 (1.20–1.84)	0.611
**Phosphate 6 months post-transplant**	0.91 (0.79–1.04)	0.83 (0.67–1.09)	0.611
**Liver**	**n = 9**	**n = 61**	
**Sex (male)**	6 (66.7%)	41 (67.2%)	1
**Age**	64.5 (58.6–68.7)	54.2 (44.0–60.1)	0.004
**BMI**	24.1 (22.4–26.0)	24.6 (21.5–28.0)	0.979
**Steroid containing immunosuppressive regimen**^¶^	7 (77.8%)	30 (49.2%)	0.157
**Diabetes mellitus**	5 (55.6%)	23 (37.7%)	0.468
**25-OHD peri-transplant**	23.5 (12.7–33.5)	29.0 (14.2–53.7)	0.499
**25-OHD 6 months post-transplant**	29.2 (14.0–62.9)	33.0 (17.7–53.9)	0.874
**1, 25-(OH)**_**2**_**D peri-transplant**	22.8 (9.4–30.1)	26.0 (15.5–35.9)	0.182
**1, 25-(OH)**_**2**_**D 6 months post-transplant**	21.6 (18.7–36.8)	30.0 (20.1–40.8)	0.330
**iPTH peri-transplant**	29.4 (16.6–103.8)	36.0 (22.7–59.2)	0.986
**iPTH 6 months post-transplant**	35.9 (26.2–43.4)	46.2 (36.5–64.1)	0.112
**CTx peri-transplant**	0.50 (0.30–1.50)	0.44 (0.24–0.74)	0.317
**CTx 6 months post-transplant**	0.78 (0.58–1.04)	0.72 (0.45–1.02)	0.467
**P1NP peri-transplant**	95.3 (92.6–186.2)	71.9 (53.6–127.1)	0.277
**P1NP 6 months post-transplant**	132.9 (65.7–185.6)	125.0 (70.2–204.0)	0.930
**eGFR peri-transplant**	55.9 (15.4–101.2)	89.5 (61.3–109.1)	0.125
**eGFR 6 months post-transplant**	63.9 (34.0–71.2)	67.9 (53.4–86.8)	0.335
**Phosphate peri-transplant**	1.25 (1.00–1.34)	1.05 (0.86–1.33)	0.397
**Phosphate 6 months post-transplant**	1.18 (1.18–1.36)	1.21 (1.09–1.39)	0.759

Numeric variables expressed as median (IQR), categorical variables as absolute numbers (frequencies).

Age reported in years

BMI (body mass index) reported in kg/m^2^

25-OHD (25-OH vitamin D) reported in nmol/l

1, 25-(OH)_2_D (1, 25-(OH)_2_ vitamin D) reported in ng/l

iPTH (intact parathyroid hormone) reported in ng/l

CTx (β-Crosslaps) reported in ng/ml

P1NP (total procollagen type 1 amino-terminal propeptide) reported in ng/ml

^¶^ Immunosuppressive regimen assessed 6 months after transplantation.

^#^ lower detection limit 7.5ng/l

* Fisher’s exact test and Wilcoxon rank-sum test were used for comparison, as appropriate.

#### Liver recipients

In 9 liver recipients fractures were observed, equaling an incidence of 23 fractures per 1000 person-years. Vertebral fractures were most common (n = 6, 66.7%), followed by 2 (22.2%) fractures of the upper extremities and 1 (11.1%) fracture of the lower extremities. Fractures occurred after a median of 274 days (IQR 171–661) following liver transplantation. Liver recipients with incident fractures were older (*P* = 0.004), but no significant differences were detected in BMI, diabetes mellitus, use of steroids for immunosuppression, phosphate levels and the assessed panel of biomarkers (Table 3). In 18 liver recipients BMD measurements were performed after transplantation. Normal BMD was detected in 5 (27.8%) liver transplant recipients, whereas in 11 (61.1%) and 2 (11.1%) patients osteopenia and osteoporosis were diagnosed, respectively. In 3 patients with incident fractures DXA scans were performed. 2 patients suffering from vertebral fractures had osteoporosis and 1 patient with vertebral fracture osteopenia.

## Discussion

In both, kidney and liver recipients, 25-OH vitamin D remained on similar levels within the first 6 months post-transplant, whereas 1, 25-(OH)_2_D was significantly higher 6 months post-transplant in kidney recipients, but not in liver recipients. Levels of iPTH dropped in kidney recipients, but stayed unchanged in liver recipients. The bone turnover markers CTx and P1NP showed a significant decrease in kidney transplant recipients and on the contrary a significant increase in liver transplant recipients within the first 6 months after transplantation.

Parallel to the excretory renal function an improvement of the hormonal capacity of the transplant was observed after kidney transplantation, indicated by the increased ratio of 1, 25-(OH)_2_D to 25-OHD 6 months post-transplant. The low level of 1, 25-(OH)_2_D might be also partly due to the assumed premenopausal status of most kidney transplant recipients shown in a subgroup analysis (S2 Table). The high peri-transplant iPTH levels, reflecting most likely secondary hyperparathyroidism in the majority of kidney recipients, decreased to significantly lower levels 6 months post-transplant. In kidney transplant recipients, elevated peri-transplant levels of CTx and P1NP indicated simultaneous ongoing bone formation and bone resorption. These contrarious processes were occurring simultaneously as indicated by the significant correlation between CTx and P1NP. The parallel elevation of these bone turnover markers at time of transplantation might reflect a state of high bone turnover, such as osteitis fibrosa, or might be, at least partly, caused by a diminished renal excretion of CTx and accumulation of the monomeric form of P1NP [24, 25]. In agreement with our data, a linear relationship between CTx and P1NP was described by Ueda et al for hemodialysis patients [26].

The considerable increase in both, CTx and P1NP, at 6 months post-transplant in liver recipients is indicative of a high bone turnover state. The reasons for these changes in bone metabolism are unclear. One explanation may be a combined effect of vitamin D deficiency, triggering an overall, albeit not significant, increase in iPTH, and deterioration of kidney function, possibly due to calcineurin-inhibitor toxicity, thus promoting incipient renal osteodystrophy. This hypothesis would be supported by the significant decrease in eGFR of liver recipients. Alternatively, the change in eGFR might be influenced by a gain in muscle mass post-transplant.

The longitudinal development of the assessed biomarkers showed major differences between kidney and liver transplant recipients in 1, 25-(OH)_2_ vitamin D, iPTH, CTx, P1NP, eGFR and phosphate levels. In kidney transplant recipients the increase in 1-position hydroxylation of vitamin D was to be expected as the decrease in iPTH due to hormonal activity of the graft. Likely, the improvement in eGFR and decrease in phosphate reflects exocrine renal function of the kidney graft. With regard to the hormonal function of the liver an increase in 25-OH vitamin D would be assumed. The avoidance of sun exposure resulting in a shortage of the precursor hormone vitamin D might be a possible explanation for the unchanged 25-OH vitamin D levels after liver transplantation. The observed decline in eGFR after liver transplantation has been reported before [27] and might be multifactorial as discussed above.

Bone turnover markers are rarely measured in clinical routine. Determination of CTx and P1NP has been encouraged as a monitoring tool in patients treated for various bone disorders [28, 29]. Previous studies have demonstrated a correlation of CTx with biopsy-proven bone resorption [30]. A further application of bone turnover markers includes cancer patients, where CTx has been shown to be a marker of bone metastases [31] and of bone involvement in multiple myeloma [32]. Beyond determination of vitamin D metabolites, iPTH and markers of bone metabolism our study adds a translational aspect characterizing the association of these markers with the most relevant clinical endpoint, i.e. incidence of fractures.

All reported fractures in our study were diagnosed by radiographs prompted by symptoms. 12.9% of liver recipients suffered from fractures. Previously published data on fracture incidence after liver transplantation show a wide variability [11, 33–38]. This imprecision might be caused by differences in diagnostic assessment (radiographs per protocol vs. radiographs triggered by symptoms), different lengths of patient follow up as well as temporal changes in immunosuppression with recent strategies favoring early steroid withdrawal. In our study liver graft recipients suffering from fractures were significantly older than recipients without fractures. This finding is in line with previous studies [11, 36].

Similar to liver transplantation, literature on fracture rates after kidney transplantation is characterized by a large variety [39]. Studies with longer observation periods indicated overall a higher fracture rate [40, 41]. We recorded fractures in 10% of kidney recipients over a median follow-up of 5.6 years. Consistent with literature most fractures in kidney recipients affected the appendicular skeleton and, in particular, the lower extremities [12, 42]. In kidney graft recipients with incident fractures 1, 25-(OH)_2_D levels were significantly lower peri-transplant, this observation waned to a trend at 6 months post-transplant. This finding highlights the importance of the biologically active vitamin D metabolite for bone health. Higher levels of iPTH and a trend of higher CTx were found in kidney recipients without incident fractures 6 months post-transplant, but not at time of transplantation. This observation might indicate that a certain level of bone resorption needs to be maintained for bone health. A longer timespan between kidney transplantation and BMD measurement showed a negative linear relationship with the T-score of the femoral neck. Time-dependent deleterious effects of immunosuppression might be at the cause of this observation. Immunosuppressive agents have been associated with abnormal bone composition. This harmful effect is best established for corticosteroids which have been linked to both, osteoporosis and fractures [43, 44]. Of note, in our study all kidney transplant recipients with incident fractures received a steroid-containing immunosuppressive regimen at least until 6 months post-transplant (vs. 87.3% of patients without incident fractures). The impact of other immunosuppressive agents on bone metabolism is less clear, e. g. conflicting results have been reported for cyclosporine A [45–47].

One main strength of our study lies in the longitudinal, uniform measurement of key variables involved in bone metabolism, including vitamin D metabolites, iPTH and the bone turnover markers CTx and P1NP, utilizing samples derived from time of transplant and 6 months after transplantation. The multicenter cohort design with participation of all Swiss transplant centers minimizes the impact of center-specific differences in post-transplant patient care.

Our study has several limitations. First, data on DXA scans and incident fractures were retrospectively collected. Second, BMD measurements were performed at the discretion of the treating physician and not according to a protocol. All analyzed DXA scans were performed post-transplant, different devices were used and scans were done at varying time points post-transplant, but timespan since transplantation is likely a crucial factor for bone loss. Baseline BMD at time of transplantation was not assessed, thus we might have missed preexisting abnormal BMD. Third, radiographs were exclusively taken, if clinically indicated, i.e. suspicion of a fracture. This approach inevitably results in missing asymptomatic fractures.

In conclusion, our data illustrates substantial alterations of bone metabolism markers in the first 6 months after transplantation. Hallmarks were a significant gain in 1, 25-(OH)_2_D combined with a decrease in iPTH, CTx and P1NP after kidney transplantation and an increase in CTx and P1NP after liver transplantation.

Future studies with longitudinal measurement of a comprehensive bone metabolism panel, including CTx and P1NP, combined with serial DXA scans could help to gain a more precise insight into the role of bone turnover markers in the transplant population.

## Supporting information

S1 Table Reference values and coefficients of variation for used laboratory assays. (DOCX)

S2 Table Comparison of vitamin D metabolites, iPTH, CTx, P1NP and phosphate between pre- and postmenopausal female transplant recipients. (DOCX)

S1 Fig Correlation of CTx and P1NP in kidney recipients and liver recipients. Line was generated corresponding to univariable linear regression.
(TIF)

S2 Fig Correlation of CTx and iPTH in kidney recipients and liver recipients. Line was generated corresponding to univariable linear regression.
(TIF)
