# Supplementary material for: Bone metabolism dynamics in the early post-transplant period following kidney and liver transplantation
Source: PLoS One. 2018 Jan 16;13(1):e0191167. doi: 10.1371/journal.pone.0191167 (PMC5770064; doi:10.1371/journal.pone.0191167)
Supplement: S1 Table — (DOCX) [file pone.0191167.s001.docx]

**Supplemental Table 1: Reference values and coefficients of variation for used laboratory assays**

| **Analysis** | **Reference values** | **Coefficients of variation*** |
| --- | --- | --- |
| **25-OH vitamin D** | All sexes and ages:  severe deficiency < 25nmol/l  deficiency ≥ 25 and < 50nmol/l  no deficiency ≥ 50nmol/l | <10% |
| **1, 25-(OH)_2_ vitamin D** | All sexes and ages: 19.9-79.3ng/l | <12.6% |
| **intact PTH** | All sexes and ages: 15.0‑65.0ng/l | <3.0% |
| **β-Crosslaps** | Male, 30-50 years: <0.58ng/ml  Male, 51-70 years: <0.7ng/ml  Male, >70 years: <0.85ng/ml  Female, premenopausal: <0.57ng/ml  Female, postmenopausal: < 1ng/ml | <1.7% |
| **total procollagen type 1 amino-terminal propeptide** | All sexes and ages: 15-59ng/ml | <3.3% |
| **Phosphate** | All sexes and ages: 0.87‑1.45mmol/l | <0.9% |

* intra- and interday imprecision for each 20 replicates of 2 levels of quality control materials over subsequent 20 days
