# Supplementary material for: Bone metabolism dynamics in the early post-transplant period following kidney and liver transplantation
Source: PLoS One. 2018 Jan 16;13(1):e0191167. doi: 10.1371/journal.pone.0191167 (PMC5770064; doi:10.1371/journal.pone.0191167)
Supplement: S2 Table — (DOCX) [file pone.0191167.s002.docx]

**Supplemental Table 2: Comparison of vitamin D metabolites, iPTH, CTx, P1NP and phosphate between pre- and postmenopausal female transplant recipients**

|  | **Premenopause^#^** | **Postmenopause^#^** | ***P**** |
| --- | --- | --- | --- |
| **kidney** | **n = 20** | **n = 8** |  |
| **25-OHD peri-transplant** | 26.1 (10.5-42.1) | 31.1 (17.1-61.9) | 0.427 |
| **25-OHD 6 months post-transplant** | 39.2 (23.5-49.4) | 43.1 (26.3-64.0) | 0.265 |
| **1, 25-(OH)_2_D peri-transplant** | 7.6 (7.5-11.9) | 15.3 (12.3-25.0) | 0.024 |
| **1, 25-(OH)_2_D 6 months post-transplant** | 39.8 (25.7-55.0) | 29.0 (23.9-34.1) | 0.238 |
| **iPTH peri-transplant** | 179.4 (66.8-365.5) | 114.6 (63.8-149.5) | 0.329 |
| **iPTH 6 months post-transplant** | 76.8 (62.7-91.6) | 53.8 (37.3-57.1) | 0.032 |
| **CTx peri-transplant** | 1.52 (0.83-1.99) | 0.63 (0.51-0.99) | 0.060 |
| **CTx 6 months post-transplant** | 0.52 (0.17-0.87) | 0.21 (0.16-0.36) | 0.253 |
| **P1NP peri-transplant** | 191.4 (85.8-252.7) | 138.2 (61.6-228.8) | 0.566 |
| **P1NP 6 months post-transplant** | 62.3 (38.4-98.0) | 29.3 (22.0-69.8) | 0.260 |
| **Phosphate peri-transplant** | 1.55 (1.28-2.16) | 1.15 (0.64-1.69) | 0.178 |
| **Phosphate 6 months post-transplant** | 0.97 (0.69-1.10) | 1.01 (0.93-1.34) | 0.387 |
| **liver** | **n = 15** | **n = 8** |  |
| **25-OHD peri-transplant** | 29.0 (15.6-52.4) | 47.9 (35.9-77.1) | 0.100 |
| **25-OHD 6 months post-transplant** | 38.7 (17.7-64.6) | 42.9 (13.5-79.8) | 0.674 |
| **1, 25-(OH)_2_D peri-transplant** | 30.3 (28.6-45.4) | 26.0 (20.0-37.5) | 0.186 |
| **1, 25-(OH)_2_D 6 months post-transplant** | 38.7 (29.3-41.2) | 19.4 (10.9-31.6) | 0.149 |
| **iPTH peri-transplant** | 41.5 (27.4-69.1) | 58.0 (24.8-97.8) | 0.728 |
| **iPTH 6 months post-transplant** | 46.5 (39.5-57.0) | 37.3 (31.0-53.5) | 0.294 |
| **CTx peri-transplant** | 0.26 (0.20-0.44) | 0.57 (0.51-0.80) | 0.026 |
| **CTx 6 months post-transplant** | 0.75 (0.41-0.96) | 0.69 (0.57-1.09) | 0.591 |
| **P1NP peri-transplant** | 59.3 (31.9-80.4) | 117.0 (77.8-199.7) | 0.101 |
| **P1NP 6 months post-transplant** | 97.6 (65.5-180.0) | 132.4 (97.3-425.5) | 0.325 |
| **Phosphate peri-transplant** | 1.15 (0.89-1.39) | 1.32 (1.00-1.43) | 0.771 |
| **Phosphate 6 months post-transplant** | 1.29 (1.20-1.36) | 1.22 (1.20-1.26) | 0.628 |

^#^Age-based assignment: <55y premenopausal, ≥55y postmenopausal

Numeric variables expressed as median (IQR).

25-OHD (25-OH vitamin D) reported in nmol/l

1, 25-(OH)_2_D (1, 25-(OH)_2_ vitamin D) reported in ng/l

iPTH (intact parathyroid hormone) reported in ng/l

CTx (β-Crosslaps) reported in ng/ml

P1NP (total procollagen type 1 amino-terminal propeptide) reported in ng/ml

Phosphate reported in mmol/l

* Wilcoxon rank-sum test was used for comparison.
