# Supplementary figures and images for: Bone metabolism dynamics in the early post-transplant period following kidney and liver transplantation
Source: PLoS One. 2018 Jan 16;13(1):e0191167. doi: 10.1371/journal.pone.0191167 (PMC5770064; doi:10.1371/journal.pone.0191167)

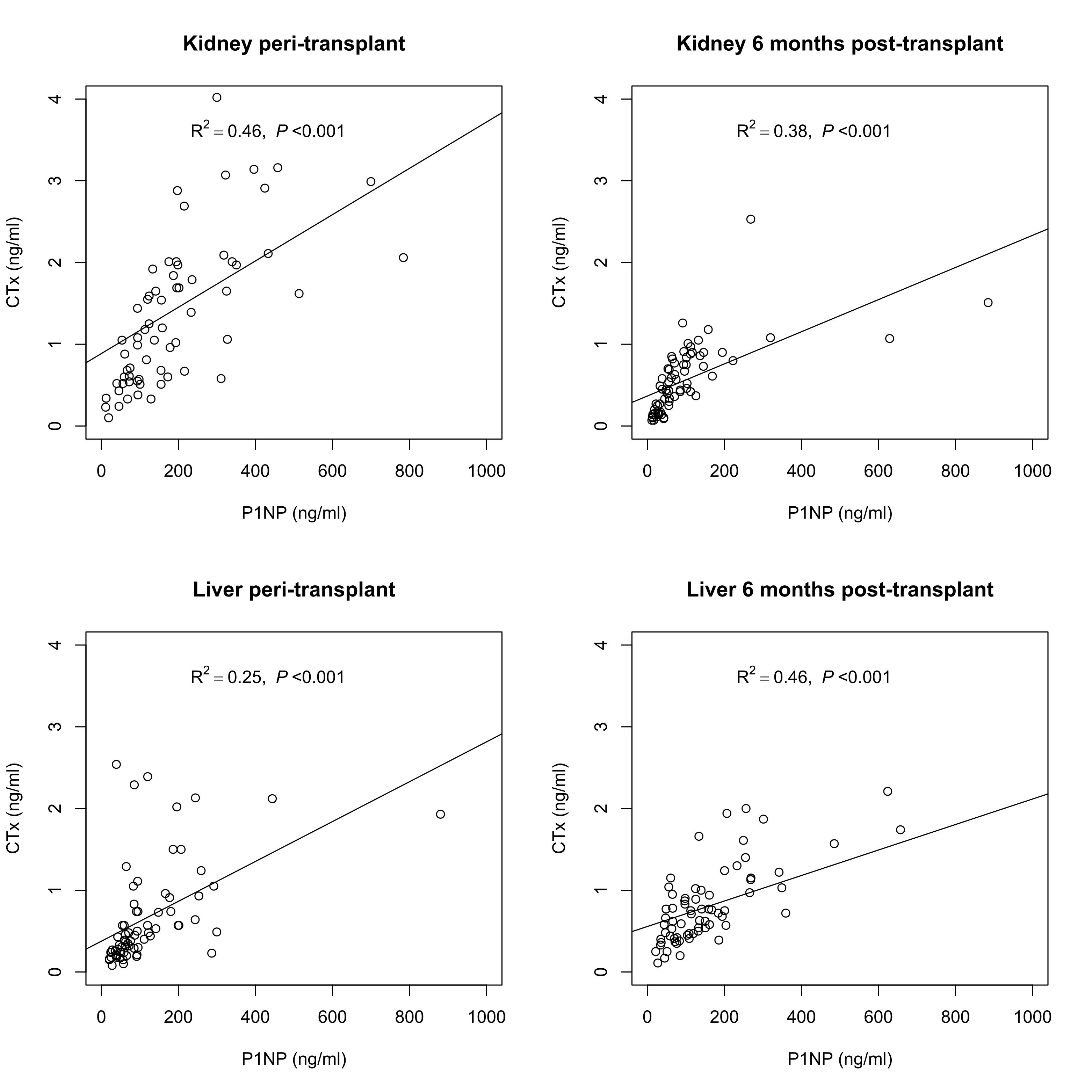

Supplement: S1 Fig — Line was generated corresponding to univariable linear regression. (TIF) [file pone.0191167.s003.tif]

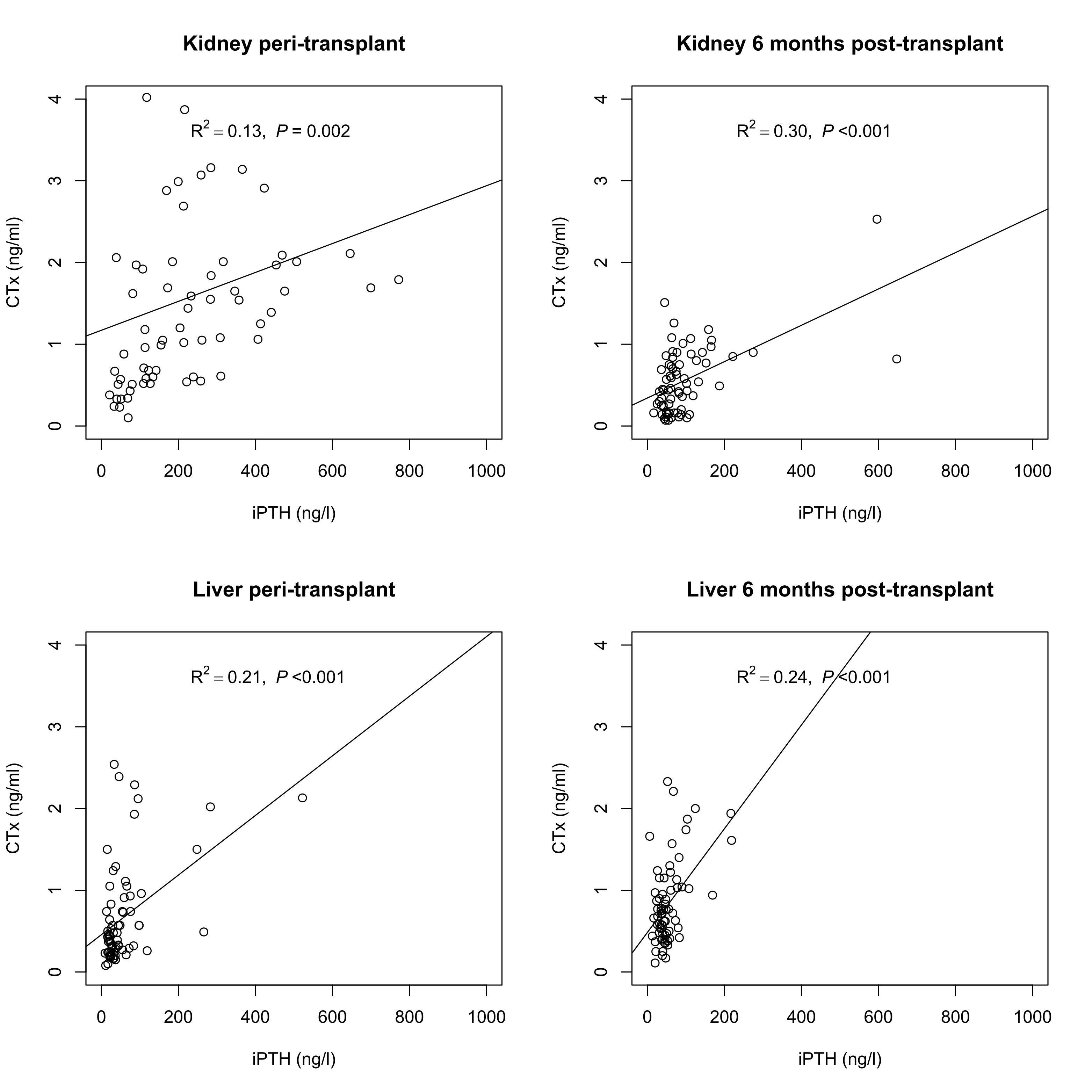

Supplement: S2 Fig — Line was generated corresponding to univariable linear regression. (TIF) [file pone.0191167.s004.tif]
